# Do Not Overlook the Vessels: A Lesson in Quadriceps Pain and Femoral Artery Embolic Myxoma

**DOI:** 10.7759/cureus.91385

**Published:** 2025-09-01

**Authors:** Chong Qi Tan, Amirzeb Aurangzeb, Mattheaus Sheng Jie Lim, Suresh Babu, Sanjay Nalachandran, Dinesh Sirisena

**Affiliations:** 1 Orthopedic Surgery, National University Hospital, Singapore, SGP; 2 Orthopedic Surgery, Changi General Hospital, Singapore, SGP; 3 Family Medicine, Singapore General Hospital, Singapore, SGP; 4 Interventional Radiology, International Orthopedic Clinic, Singapore, SGP; 5 General Surgery, Gleneagles Hospital Singapore, Singapore, SGP; 6 Sports Medicine, National University Health System, Singapore, SGP

**Keywords:** arterial occlusion, #myxoma, orthopaedic sports medicine, primary care sports medicine, quadriceps pain

## Abstract

Quadriceps pain is a common musculoskeletal complaint, often attributed to exercise-induced strain, muscle injury, or overuse in active individuals. However, when pain persists disproportionately to exertion or occurs with subtle vascular signs, it may signal more sinister pathology.

This is a case of an otherwise healthy 35-year-old male amateur athlete whose subacute left quadriceps pain ultimately revealed an unusual vascular emergency. The patient reported several days of worsening anterior thigh discomfort during sport, progressing to persistent soreness at rest. Upon further history and examination, concerning features such as reduced distal pulses, a femoral bruit, and disproportionate pain-to-activity history were present, increasing the suspicion of a vascular cause of the pain.

This case underscores the importance of a comprehensive clinical assessment, with thorough history-taking and careful physical examination, followed by appropriate investigations. Consideration of broad differentials is key to accurate diagnosis and timely intervention. Early recognition of vascular compromise and prompt specialist referral can prevent delayed diagnosis and optimize patient outcomes in these potentially limb-threatening presentations.

## Introduction

Quadriceps pain is a frequent complaint in orthopedic and sports medicine practice, typically attributed to muscle strains, overuse injuries, or tendinopathies [1,2]. While most cases resolve with conservative measures, persistent or disproportionate pain should prompt consideration of less common, yet potentially limb-threatening, vascular pathologies [3]. Although uncommon in a young population, vascular causes of thigh pain, such as peripheral arterial disease, deep vein thrombosis, or compartment syndrome, are rare but critical to recognize early [4]. Delayed diagnosis can lead to irreversible muscle ischemia, limb loss, or life-threatening complications [5].

We present an unusual case of a young athlete whose seemingly benign quadriceps pain masked an underlying arterial occlusion caused by emboli of myxomatous origin. This case highlights the critical importance of thorough clinical evaluation and maintaining a broad differential diagnosis in practice.

## Case presentation

A 35-year-old healthy Chinese male presented with exercise-induced left lower limb symptoms. He first experienced cramping and pain in his left lower limb while playing squash that persisted for several days. There was no preceding trauma or increased intensity of physical exertion prior to the onset of symptoms. Since this initial episode, he reported recurrent exertional quadriceps pain, exacerbated by stair climbing and relieved with rest. The pain was not radiating and was localized to the quadriceps. He did not have any lower back, hip, or knee pain. He was otherwise able to perform daily activities with no limitations. He had no family history of coagulopathies. Due to his symptoms, he had previously sought treatment from physical therapy specialists who provided soft tissue therapy and rehabilitation exercises to no avail.

On physical examination, the left lower limb demonstrated full range of motion without pain during hip flexion, during knee extension, or on passive stretch. There was no reproducible pain or focal tenderness on palpation of the quadriceps muscles. However, vascular assessment revealed reduced pulses in the dorsalis pedis, tibialis posterior, popliteal, and femoral arteries, with an audible femoral bruit. Capillary refill time was otherwise unremarkable.

Table 1 shows the Doppler ultrasound findings of the left lower limb, which revealed a mildly hyper-echoic ovoid structure within the lumen of the left common femoral artery-superficial artery (CFA-SFA) junction measuring 1.7x 0.7cm. The waveform at the area of thrombus/embolus was monophasic, and peak systolic velocity was 262 cm/s. This was suspicious for a left CFA-SFA junction thrombus/embolus that was causing severe stenosis.

**Table 1:** Doppler ultrasound of the left lower limb PSV, peak systolic velocity

Artery	Waveform	PSV (cm/s)
Common femoral artery	Monophasic (at area of thrombus)	262
Superficial femoral artery	Biphasic	14
Profunda femoral artery	Biphasic	59
Popliteal artery	Biphasic	13
Anterior tibial artery	Monophasic	11
Peroneal artery	Monophasic	11
Posterior tibial artery	Monophasic	12
Dorsalis pedis artery	Monophasic	7

**Figure 1:**
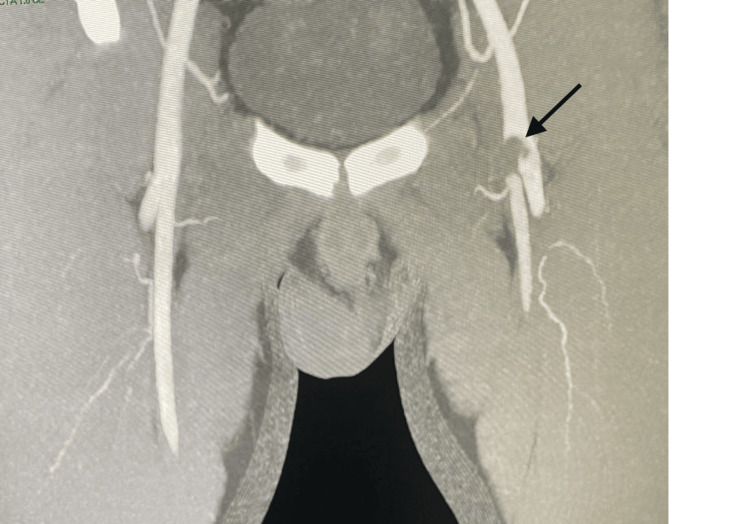
CT angiogram CT, computed tomography

An urgent computed tomography (CT) angiogram was then performed for further evaluation and to confirm the diagnosis. Figure 1 shows the CT angiogram findings of a thrombus present at the left femoral bifurcation, extending slightly into the superficial femoral artery and the profunda artery. No aneurysm or dissection was seen in the femoral artery.

**Figure 2:**
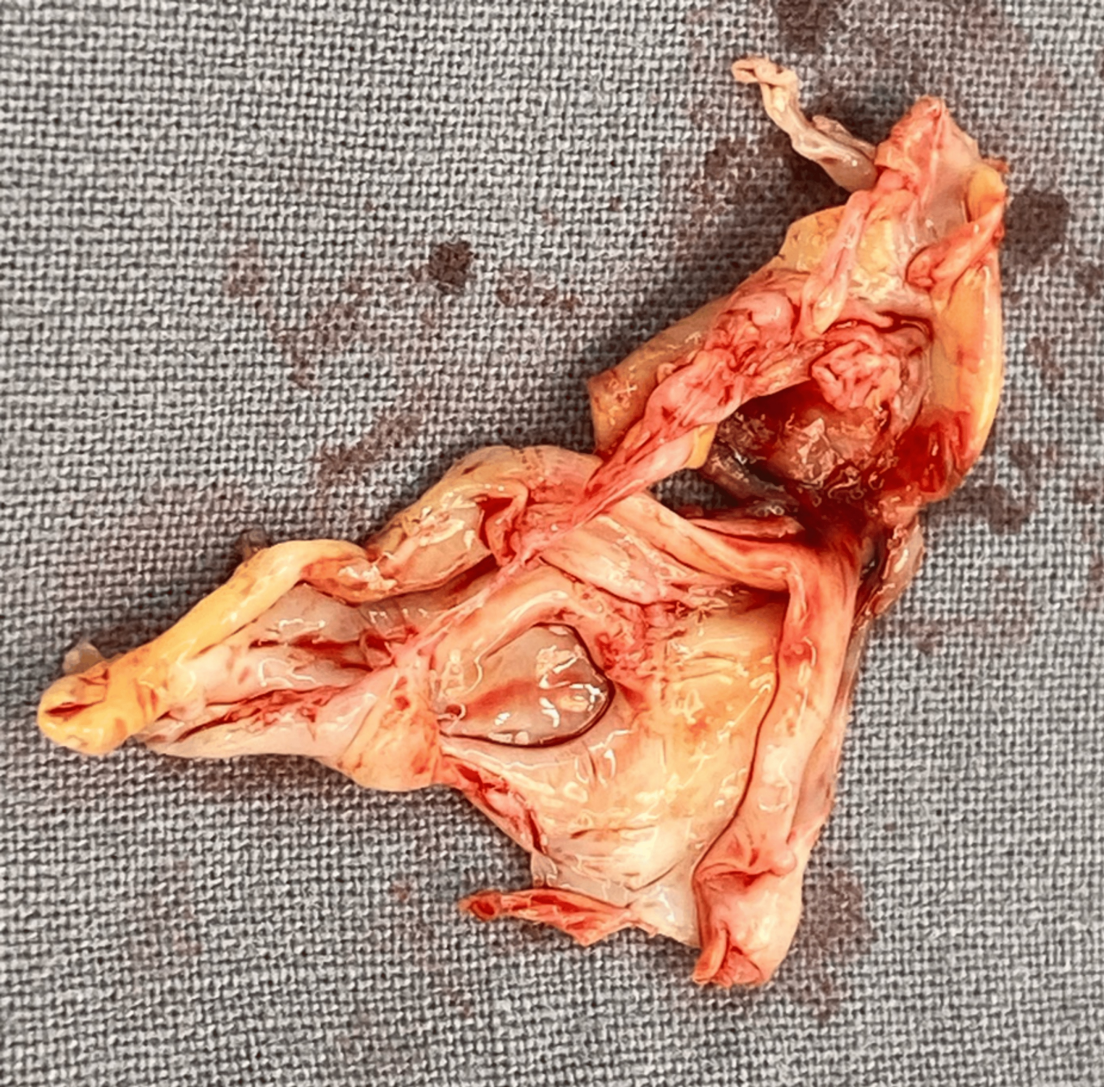
Image of the thrombus

An urgent vascular consultation was made, and the patient subsequently underwent a femoral endarterectomy to remove the thrombus (Figure 2) to restore vascular flow to the left lower limb.

Subsequent histopathological analysis of the thrombus uncovered an even more surprising feature: extensive myxoid change, a finding commonly associated with cardiac myxomas. Yet, extensive cardiac evaluation with two separate transthoracic echocardiograms revealed no definitive identifiable cardiac source.

Post-operatively, there were no significant complications, distal pulses were comparable to the contralateral side, and the patient was able to progressively return to physical activity after a period of rehabilitation.

## Discussion

Quadriceps pain is an exceedingly common complaint [6] among active individuals, frequently attributed to benign musculoskeletal etiologies such as muscle strains [7,8] , overuse injuries [9], exercise-induced myositis, or delayed-onset muscle soreness. This prevalence often leads clinicians to adopt a conservative diagnostic approach, particularly in young, healthy patients without traditional risk factors for vascular disease. However, our case underscores a critical clinical lesson: even the most routine presentations demand a broad differential diagnosis when symptoms persist or exhibit atypical features, even in low-risk populations such as in this case. The patient’s initially unremarkable exertional thigh pain ultimately revealed a limb-threatening femoral artery thrombus, challenging conventional diagnostic assumptions.

Pitfalls of cognitive biases in clinical practice

This case illustrates how cognitive biases can delay critical diagnoses. Utilizing heuristic shortcuts based on the patient’s demographic (age, athletic background) and prevalence of musculoskeletal injury in this age group led to the initial erroneous diagnosis of quadriceps strain. This diagnostic error was fortunately corrected when persistent symptoms prompted a thorough re-evaluation, where key red flag signs were then picked up, such as disproportionate pain and subtle vascular signs (absent pulses, bruit). Such pitfalls underscore the need for disciplined, systematic approaches to counteract inherent cognitive biases, such as maintaining broad differential diagnoses (Table 2), employing checklists to ensure key findings (pulses, neurologic signs) are not missed, and scheduling deliberate "diagnostic time-outs" when treatment fails. By acknowledging our innate tendency to favor pattern recognition and initial impressions, and thereby implementing structured safeguards, clinicians can better navigate the tension between efficiency and thoroughness, ensuring that atypical presentations (such as in this case, where a vascular pathology masqueraded as a strain) receive timely consideration before irreversible harm occurs.

**Table 2:** Common differential diagnoses of quadriceps pain (non-exhaustive) SLR, straight leg raise; CK, creatine kinase

Category	Key Differentiators	Possible Diagnoses	Study
Musculoskeletal	• Localized tenderness	• Quadriceps strain	[1,3]
	• Tendinopathy	• Stress fracture	
	• Stress fracture		
	• Pain with muscle activation		
	• No vascular compromise		
Vascular	• Absent/diminished pulses	• Peripheral artery disease	[10–15]
	• Bruit on auscultation	• Popliteal artery entrapment	
	• Limb swelling/discoloration	• Deep vein thrombosis	
	• Capillary refill >2 sec		
Neurological	• Radiating pain pattern	• Lumbar radiculopathy (L2–L4)	[16,17]
	• Sensory changes		
	• Normal pulses		
	• Positive SLR test		
Systemic	• Elevated CK (>1,000 U/L)	• Myositis	[18,19]
	• Systemic symptoms (fever, malaise)	• Rhabdomyolysis	
	• Dark urine		

Critical role of vascular examination and imaging

A systematic vascular examination consisting of pulse palpation (radial, femoral, popliteal, dorsalis pedis, and posterior tibial), auscultation for bruits, and capillary refill assessment should be mandatory in all limb pain evaluations, regardless of presumed musculoskeletal etiology. This case illustrates how meticulous vascular evaluation revealed critical findings (absent distal pulses and femoral bruit) that contradicted the initial diagnosis and necessitated urgent vascular imaging. This is vital as missed vascular assessments may result in devastating diagnostic delays, particularly in limb-threatening ischemia. While peripheral arterial disease is rare in young athletes, clinicians must maintain high suspicion for alternative vascular pathologies including popliteal artery entrapment [10,11], acute thrombotic occlusion, and vasculitis processes.

Key features warranting vascular assessment include claudication-like pain (exertional discomfort resolving with rest) [12], rest pain (suggesting critical ischemia) [13,14], pulse deficits or asymmetries [15], bruits or thrill on palpation [12], and delayed capillary refill or cool extremities [12,13].

This systematic approach ensures timely identification of potentially limb-threatening vascular conditions that might otherwise be overlooked in athletic populations. When vascular compromise is suspected, prompt imaging (Doppler ultrasound, CT angiography) is essential. Early vascular surgery consultation enabled successful intervention, highlighting how cross-specialty collaboration improves outcomes [20]. Clinicians should maintain a low threshold for cross-specialty consultation when clinical findings deviate from typical musculoskeletal patterns.

Role of histopathology in vascular cases

This case underscores the critical need for systematic histopathological analysis of arterial thrombi, especially in three key scenarios: (1) young patients lacking traditional cardiovascular risk factors, (2) cases of idiopathic arterial occlusion, and (3) when clinical features raise suspicion for neoplastic or inflammatory pathologies.

The identification of myxomatous change in our patient's thrombus significantly redirected clinical management, prompting additional investigations [21]. However, this subsequently revealed a perplexing diagnostic dilemma as comprehensive echocardiographic evaluation failed to detect a primary cardiac tumor. This discrepancy presents several possibilities such as complete tumor embolization, an extra-cardiac myxoma source, or limitations with echocardiography. The diagnostic uncertainty was further compounded by the patient's subsequent refusal of recommended advanced imaging (cardiac MRI, PET-CT) and longitudinal monitoring. Despite these challenges, clinicians must emphasize the importance of complete diagnostic evaluation to guide targeted therapy, as identification of the underlying pathology is essential for determining appropriate long-term management strategies.

## Conclusions

This rare case reinforces the importance of clinical vigilance during the evaluation of common clinical presentations such as quadriceps pain. While muscular causes are most often benign, persistent or atypical features such as disproportionate pain or subtle vascular signs warrant a thorough, systematic assessment. Clinicians must resist anchoring bias and pursue methodical evaluation, as it can delay the recognition of rarer but more serious conditions such as arterial occlusion in this case.
